# Changes in Emergency Department Utilization in Vulnerable Populations After COVID-19 Shelter-in-Place Orders

**DOI:** 10.7759/cureus.60556

**Published:** 2024-05-18

**Authors:** Philip R Wang, Akhil Anand, James F Bena, Shannon Morrison, Jeremy Weleff

**Affiliations:** 1 Department of Psychiatry, Cleveland Clinic Lerner College of Medicine of Case Western Reserve University, Cleveland, USA; 2 Department of Psychiatry and Psychology, Neurological Institute, Cleveland Clinic, Cleveland, USA; 3 Department of Quantitative Health Sciences, Cleveland Clinic, Cleveland, USA; 4 Department of Psychiatry, Yale School of Medicine, New Haven, USA

**Keywords:** z codes, emergency department utilization, substance use disorders, mental health, social determinants of health

## Abstract

Objectives: This study aims to compare emergency department (ED) utilization and admission rates for patients with a history of mental health (MH) disorders, substance use disorders (SUDs), and social determinants of health (SDOH) before and after implementing COVID-19 shelter-in-place (SIP) orders.

Methods: This was a retrospective, multicenter study leveraging electronic medical record (EMR) data from 20 EDs across a large Midwest integrated healthcare system from 5/2/2019 to 12/31/2019 (pre-SIP) and from 5/2/2020 to 12/31/2020 (post-SIP). Diagnoses were documented in the patient's medical records. Poisson and logistic regression models were used to evaluate ED utilization and admission rate changes.

Results: A total of 871,020 ED encounters from 487,028 unique patients were captured. Overall, 2,572 (0.53%) patients had a documented Z code for SDOH. Patients with previously diagnosed MH disorders or SUDs were more likely to seek ED care after the SIP orders were implemented (risk ratio (RR): 1.20, 95% confidence interval (CI): 1.18-1.22, p<0.001), as were patients with SDOH (RR: 2.37, 95% CI: 2.19-2.55, p<0.001). Patients with both previously diagnosed MH disorders or SUDs and a documented SDOH had even higher ED utilization (RR: 3.31, 95% CI: 2.83-3.88, p<0.001) than those with either condition alone. Patients with MH disorders and SUDs (OR: 0.89, 95% CI: 0.86-0.92, p<0.001) or SDOH (OR: 0.67, 95% CI: 0.54-0.83, p<0.001) were less likely to be admitted post-SIP orders, while patients with a history of diseases of physiologic systems were more likely to be admitted.

Conclusion: Vulnerable populations with a history of MH disorders, SUDs, and SDOH experienced increased ED utilization but a lower rate of hospital admissions after the implementation of SIP orders. The findings highlight the importance of addressing these needs to mitigate the impact of public health crises on these populations.

## Introduction

Patients with mental health (MH) disorders and substance use disorders (SUDs) face significant challenges in accessing healthcare equitably [1]. In addition, social determinants of health (SDOH) profoundly impact healthcare utilization and delivery, quality of life, and mortality. Identifying these patients for targeted interventions is crucial [2]; however, inconsistent and non-standardized documentation of SDOH across healthcare systems hinders public health surveillance efforts of this population. To address this issue, the Centers for Medicare and Medicaid Services and other groups have proposed using Z codes to standardize the documentation of social needs, but the utilization of these codes remains limited [3,4].

The COVID-19 pandemic has exacerbated health disparities and disproportionately affected individuals with MH disorders, SUDs, and SDOH [5]. Shelter-in-place (SIP) orders were enacted by many governments to encourage residents to stay at home to prevent the spread of COVID-19. While research has shown that SIP orders have led to increased ED visits for mental health and substance use reasons [6-8], few studies have examined the impact on ED utilization and admissions in individuals with a prior history of MH disorders or SUDs. Furthermore, the effects of the COVID-19 pandemic on ED utilization and admissions by patients with SDOH have been poorly studied thus far. The primary aim of this study is to investigate the changes in ED utilization and hospital admission rates among vulnerable populations with a history of MH disorders, SUDs, and SDOH before and after SIP orders across a large integrated healthcare system. A secondary aim was to characterize the frequency of utilization of Z codes to identify patients with SDOH in the ED. This article was previously posted to the medRxiv preprint server on October 26, 2023 [9].

## Materials and methods

Study design

This retrospective, cross-sectional analysis collected electronic medical record (EMR) data to analyze ED visits from patients with MH disorders, SUDs, and SDOH. Inclusion criteria included patients above the age of 18 who visited any of 20 EDs ranging from large hospital-associated EDs to freestanding EDs across a large Midwest integrated healthcare system in Ohio from 5/2/2019 to 12/31/2019 ("pre-SIP order" time period) and from 5/2/2020 to 12/31/2020 ("post-SIP order" time period). The sole exclusion criteria were patients under the age of 18. This research was approved by the Cleveland Clinic Institutional Review Board under study number 20-974.

Data collection

Demographics and diagnoses were extracted from the EMR. MH disorders and SUDs were identified using the International Classification of Diseases, 10th revision (ICD-10) codes; similarly, SDOH were identified in patients' medical histories using respective Z codes in the ICD-10. Historical use of these codes means that these diagnoses and SDOH were documented in the patient's chart at any time and not solely reliant on ED encounter documentation.

Statistical analysis

Poisson regression models were used to calculate absolute risk ratios (RR) to compare the total number of cases between the pre- and post-SIP periods. Logistic regression models were used to evaluate changes in admission rates between the two periods, and odds ratios (OR) were calculated.

## Results

Patient demographics

A total of 871,020 ED encounters from 487,028 unique patients were captured; 473,449 (54%) visits occurred in 2019, and 397,571 (46%) were in 2020. Our cohort was mostly White (65.4%), female (53.8%), and privately insured (68.9%) and had a mean age of 46.0±24.0. Overall, 2,572 (0.53%) patients had a documented Z code for SDOH. The most coded SDOH in ED encounters was problems related to housing and economic circumstances, followed by other problems related to the primary support group and other psychosocial circumstances. Compared to patients without SDOH, patients with a coded SDOH were more likely to be Black, younger, identified as male, and on government or self-pay insurance (Table 1).

**Table 1:** Demographics of patients with and without social determinants of health ^a^Pearson's chi-square test ^b^Satterthwaite t-test Bolded p-values indicate ones below the significance level α=0.05. SD: standard deviation, ED: emergency department

Characteristic	Total (N=487,028)	No coded social determinant of health (N=484,456)	Coded determinants of health (N=2,572)	p-value
Race (number (%))				<0.001^a^
White	306,850 (65.4)	305,708 (65.5)	1,142 (47.6)	
Black	132,895 (28.3)	131,905 (28.3)	990 (41.3)	
Multiracial/multicultural	22,721 (4.8)	22,485 (4.8)	236 (9.8)	
Asian	4,549 (0.97)	4,524 (0.97)	25 (1.04)	
American Indian/Alaskan Native	611 (0.13)	609 (0.13)	2 (0.08)	
Sex (number (%))				<0.001a
Female	262,222 (53.8)	261,028 (53.9)	1,194 (46.4)	
Male	224,754 (46.2)	223,376 (46.1)	1,378 (53.6)	
Hispanic race (number (%))				0.027^a^
Hispanic	36,939 (7.8)	36,717 (7.8)	222 (8.9)	
Not Hispanic	438,901 (92.2)	436,641 (92.2)	2,260 (91.1)	
Age (mean±SD)	46.0±24.0	46.1±23.9	28.7±26.0	<0.001^b^
Admitted from ED (number (%))	111,010 (22.8)	110,491 (22.8)	519 (20.2)	0.001^a^
Insurance (number (%))				<0.001^a^
Government (Medicare/Medicaid) + self-pay	151,708 (31.1)	150,620 (31.1)	1,088 (42.3)	
Private + others including CCHS	335,320 (68.9)	333,836 (68.9)	1,484 (57.7)	

ED utilization rates before and after shelter-in-place orders

Compared to before the implementation of SIP orders, patients with previously diagnosed MH disorders or SUDs were more likely to seek ED care after the SIP orders were put in place (RR: 1.20, 95% confidence interval (CI): 1.18-1.22, p<0.001) (Table 2). Among patients with SDOH, those with a history of problems related to the primary support group (RR: 2.89, 95% CI: 2.54-3.29) or housing and economic circumstances (RR: 2.50, 95% CI: 2.23-2.80) were more likely to present to the ED after SIP orders. Patients with both previously diagnosed MH disorders or SUDs and a documented SDOH (RR: 3.31, 95% CI: 2.83-3.88, p<0.001) had even higher ED utilization than those with either condition alone.

**Table 2:** ED visits of categories of historical diagnoses before and after shelter-in-place orders MH: mental health, SUD: substance use disorder, SDOH: social determinant of health, ED: emergency department Bolded p-values indicate ones below the significance level α=0.05.

Measure	Before shelter-in-place orders (5/2/2019-12/31/2019) (number (%))	After shelter-in-place orders (5/2/2020-12/31/2020) (number (%))	Absolute change relative risk	p-value
Overall MH disorders, SUDs, and SDOH diagnoses				
Mental health disorders	19,394 (4.1)	23,100 (5.8)	1.19 (1.17, 1.21)	<0.001
Substance use disorders	15,420 (3.3)	19,444 (4.9)	1.26 (1.23,1.29)	<0.001
Mental health or substance use disorders	32,320 (6.8)	38,711 (9.7)	1.20 (1.18,1.22)	<0.001
Social determinants of health	955 (0.20)	2,260 (0.57)	2.37 (2.19,2.55)	<0.001
Mental health or substance use disorders and social determinants of health	200 (0.04)	662 (0.17)	3.31 (2.83,3.88)	<0.001
Mental, behavioral, and neurodevelopmental disorders				
Schizophrenia, schizotypal, delusional, and other non-mood psychotic disorders	3,175 (0.67)	4,464 (1.1)	1.41 (1.34,1.47)	<0.001
Mood (affective) disorders	6,983 (1.5)	7,242 (1.8)	1.04 (1.00,1.07)	0.03
Anxiety, dissociative, stress-related, somatoform, and other non-psychotic mental disorders	7,515 (1.6)	9,735 (2.4)	1.30 (1.26,1.33)	<0.001
Suicidal ideation	2,140 (0.45)	2,366 (0.59)	1.11 (1.04,1.17)	<0.001
Suicide attempt and self-inflicted harm	4,153 (0.88)	4,070 (1.02)	0.98 (0.94,1.02)	0.36
Substance use disorders				
Alcohol-related disorders	7,247 (1.5)	8,341 (2.1)	1.15 (1.12,1.19)	<0.001
Opioid-related disorders	1,074 (0.23)	1,239 (0.31)	1.15 (1.06,1.25)	<0.001
Cannabis-related disorders	1,263 (0.27)	2,362 (0.59)	1.87 (1.75,2.00)	<0.001
Opioid-specific overdose	473 (0.10)	329 (0.08)	0.70 (0.60,0.80)	<0.001
Social determinants of health				
Problems related to housing and economic circumstances	420 (0.09)	1,051 (0.26)	2.50 (2.23,2.80)	<0.001
Problems related to social environment	60 (0.01)	121 (0.03)	2.02 (1.48,2.75)	<0.001
Other problems related to primary support group, including family circumstances	310 (0.07)	897 (0.23)	2.89 (2.54,3.29)	<0.001
Problems related to other psychosocial circumstances	112 (0.02)	157 (0.04)	1.40 (1.10,1.79)	0.006
Transgender status	11 (0.00)	35 (0.01)	3.18 (1.62,6.26)	<0.001

ED admission rates before and after shelter-in-place orders

Compared to pre-SIP, patients with a previously diagnosed MH disorders or SUDs (OR: 0.89, 95% CI: 0.86-0.92, p<0.001) or any documented SDOH (OR: 0.67, 95% CI: 0.54-0.83, p<0.001) were less likely to be admitted to the hospital after presenting to the ED post-SIP orders (Table 3). Of the SDOH diagnosis categories, only patients with problems related to their primary support group and those with problems related to other psychosocial circumstances were significantly less likely to be admitted. The difference in admission rate pre- and post-SIP orders in patients with both MH disorders/SUDs and SDOH did not reach significance (OR: 0.65, 95% CI: 0.42-1.02, p=0.061).

**Table 3:** Admission rates of categories of historical diagnoses before and after shelter-in-place orders MH: mental health, SUD: substance use disorder, SDOH: social determinant of health Bolded p-values indicate ones below the significance level α=0.05.

Measure	Before shelter-in-place orders (5/2/2019-12/31/2019)		After shelter-in-place orders (5/2/2020-12/31/2020)		Odds ratio	p-value
	Encounters (number)	Admissions (number (%))	Encounters (number)	Admissions (number (%))		
Overall MH disorders, SUDs, and SDOH diagnoses						
Mental health disorders	19,374	7,244 (37.4)	23,087	8,140 (35.3)	0.91 (0.88, 0.95)	<0.001
Substance use disorders	15,398	5,653 (36.7)	19,425	6,453 (33.2)	0.86 (0.82, 0.90)	<0.001
Mental health or substance use disorders	32,282	11,851 (36.7)	38,680	13,174 (34.1)	0.89 (0.86, 0.92)	<0.001
Social determinants of health	955	155 (16.2)	2,259	260 (11.5)	0.67 (0.54, 0.83)	<0.001
Behavior health or substance use and social and economic disadvantage	200	32 (16.0)	662	73 (11.0)	0.65 (0.42, 1.02)	0.061
Mental, behavioral, and neurodevelopmental disorders						
Schizophrenia, schizotypal, delusional, and other non-mood psychotic disorders	3,171	1,152 (36.3)	4,462	1,481 (33.2)	0.87 (0.79, 0.96)	0.004
Mood disorders	6,972	3,064 (43.9)	7,236	3,212 (44.4)	1.02 (0.95, 1.09)	0.60
Anxiety, dissociative, stress-related, somatoform, and other non-psychotic mental disorders	7,511	2,207 (29.4)	9,726	2,501 (25.7)	0.83 (0.78, 0.89)	<0.001
Suicidal ideation	2,139	582 (27.2)	2,366	824 (34.8)	1.43 (1.26, 1.62)	<0.001
Suicide attempt and self-inflicted harm	4,150	908 (21.9)	4,065	852 (21.0)	0.95 (0.85, 1.05)	0.31
Substance use disorders						
Alcohol-related disorders	7,237	2,630 (36.3)	8,336	3,098 (37.2)	1.04 (0.97, 1.11)	0.29
Opioid-related disorders	1,074	428 (39.9)	1,238	461 (37.2)	0.90 (0.76, 1.06)	0.20
Cannabis-related disorders	1,261	319 (25.3)	2,361	587 (24.9)	0.98 (0.83, 1.14)	0.77
Opioid-specific overdose	471	47 (10.0)	329	38 (11.6)	1.18 (0.75, 1.85)	0.48
Social determinants of health						
Problems related to housing and economic circumstances	420	52 (12.4)	1,051	119 (11.3)	0.90 (0.64, 1.28)	0.57
Problems related to social environment	60	36 (60.0)	120	69 (57.5)	0.90 (0.48, 1.69)	0.75
Other problems related to primary support group, including family circumstances	310	18 (5.8)	897	25 (2.8)	0.47 (0.25, 0.86)	0.016
Problems related to other psychosocial circumstances	112	46 (41.1)	157	46 (29.3)	0.59 (0.36, 0.99)	0.046
Transgender status	11	5 (45.5)	34	13 (38.2)	0.74 (0.19, 2.93)	0.67

## Discussion

The effect of shelter-in-place orders on vulnerable patient populations

The COVID-19 pandemic has underscored the importance of addressing social determinants of health, which are critical in shaping an individual's health status and access to care. Our analysis of ED presentation and admission rates pre- and post-SIP orders found that patients with previously diagnosed MH disorders or SUDs and those with a documented SDOH were more likely to present to the ED post-SIP. Prior studies that analyzed MH and substance use-related ED visits demonstrate increased visit rates for these conditions as a primary diagnosis or chief complaint [6-8]. This study extends previous work by demonstrating that SIP orders significantly impacted vulnerable patients with a history of MH disorders, SUDs, and SDOH.

Moreover, our analysis revealed that patients with co-occurring MH disorders/SUDs and SDOH had the highest increase in ED visits following SIP orders. These patients may have faced increased difficulties in accessing care and maintaining their overall health due to the closure or reduction of community-based resources [10] and the exacerbation of existing socioeconomic inequities during the pandemic [5]. Individuals with multiple disadvantages are at a significantly higher risk of premature mortality from avoidable causes than those with a single disadvantage [11], highlighting the urgent need for tailored interventions that address the complex interplay of social, economic, and mental health-related factors contributing to poor health outcomes among these populations. Furthermore, patients with MH disorders/SUDs or SDOH were less likely to be admitted to the hospital after presenting to the ED. While our study was not designed to examine the reasons behind these disparities, possible explanations include inadequate inpatient resources for patients with MH disorders/SUDs and SDOH or healthcare professional biases, as well as structural changes of healthcare resources being shunted to emergent medical care beds.

Low documentation of social determinants of health in patients visiting the ED

Our study found that only 0.53% of ED patients had a documented Z code for SDOH before and during early COVID-19, which is lower than other rates previously reported [3,4,12]. Molina et al. [13] estimated that 1.21% of ED visits have a coded SDOH; however, our paper analyzed SDOH coding at the patient level rather than the visit level, which may explain the differences observed. The usage of Z codes to identify patients with SDOH has repeatedly been shown to be underutilized, and our results likely underestimate the number of patients with critical SDOH seeking care at EDs [14]. Future studies could consider using natural language processing models to analyze provider notes to identify patients with SDOH who do not have a documented Z code. Identifying patients with SDOH is crucial because they are more likely to experience adverse health outcomes and increased ED utilization [15,16]. Coding SDOH is currently not reimbursable, which is likely reflected in its low adoption rate; however, documentation of SDOH can substantiate patient complexity and reveal trends in healthcare utilization. For instance, if hospital systems accurately track SDOH, they can identify Z codes associated with higher readmission rates and allocate appropriate resources to improve patient care and outcomes. In the future, Z codes could play a role in determining payment rates and risk adjustment, but consistent documentation of SDOH is necessary [17].

Limitations

Our results should be interpreted in the context of their limitations. First, the retrospective design of our study limits our ability to draw causal conclusions. Second, our data comes from one large integrated healthcare system, which may differ from other healthcare systems or geographic regions. Nonetheless, our study includes a large and diverse patient population from different levels of care within the state. Third, our findings may be influenced by institution-specific documentation practices that could affect SDOH coding rates and limit the generalizability of our findings. Finally, our analysis relied on patients' past medical history documentation in the EMR, which may not reflect their current diagnoses at the time of their ED visit. However, given the time constraints of the ED, we considered capturing diagnoses from past medical history the best way to represent our sample's health status.

## Conclusions

Our study highlights the impact of the COVID-19 pandemic on vulnerable patient populations with a history of MH disorders, SUDs, and SDOH. Our findings demonstrate that these patients were more likely to present to the ED but less likely to be admitted following SIP orders, indicating a need for tailored interventions that address the complex interplay of socioeconomic and mental health-related factors contributing to poor health outcomes among these populations. The findings highlight the need for greater standardization and consistency in documenting SDOH and addressing these factors in healthcare delivery.
